# Anti-S and Anti-N Antibody Responses of COVID-19 Vaccine Recipients

**DOI:** 10.3390/vaccines11091398

**Published:** 2023-08-22

**Authors:** Abdel-Ellah Al-Shudifat, Mohammad Al-Tamimi, Rand Dawoud, Mohammad Alkhateeb, Amel Mryyian, Anas Alahmad, Manal M Abbas, Arwa Qaqish

**Affiliations:** 1 Department of Internal and Family Medicine, Faculty of Medicine, The Hashemite University, Zarqa 13133, Jordan; abdel-ellah@hu.edu.jo; 2 Department of Microbiology, Pathology and Forensic Medicine, Faculty of Medicine, The Hashemite University, Zarqa 13133, Jordan; mohammad.altamimi@hu.edu.jo; 3 Faculty of Medicine, The Hashemite University, Zarqa 13133, Jordan; randdawood123@gmail.com (R.D.); mohdoalkhateeb@hotmail.com (M.A.); amelmryan@gmail.com (A.M.); anasfayad160@yahoo.com (A.A.); 4 Department of Internal Medicine, King Hussein Cancer Center, Amman 11941, Jordan; 5 Department of Medical Laboratory Sciences, Faculty of Allied Medical Sciences, Al-Ahliyya Amman University, Amman 19328, Jordan; m.abbas2@ammanu.edu.jo; 6 Pharmacological and Diagnostic Research Lab, Al-Ahliyya Amman University, Amman 19328, Jordan; 7 Department of Biology and Biotechnology, Faculty of Science, The Hashemite University, Zarqa 13133, Jordan; 8 Department of Cellular Therapy and Applied Genomics, King Hussein Cancer Center, Amman 11941, Jordan

**Keywords:** COVID-19 vaccines, Pfizer–BioNTech, Sinopharm, AstraZeneca, antibodies, anti-S, anti-N

## Abstract

The long-term immunoglobulin responses of COVID-19 vaccinations is important to determine the efficacy of these vaccinations. This study aimed to investigate and compare the long-term immunoglobulin response of COVID-19 vaccination recipients, using anti-S IgG, anti-N IgG, and IgM titer levels. This study included 267 participants, comprising individuals who tested positive for COVID-19 through PCR testing (*n* = 125), and those who received the Pfizer (*n* = 133), Sinopharm (*n* = 112), AstraZeneca (*n* = 20), or Sputnik (*n* = 2) vaccines. Female participants comprised the largest share of this study (*n* = 147, 55.1%). This study found that most participants had positive IgG antibodies, with 96.3% having anti-S IgG and 75.7% having anti-N IgG. Most participants (90.3%) tested negative for anti-N IgM antibodies. Sinopharm-vaccinated individuals exhibited a notably lower rate of positive anti-S IgG (93.8%) and a significantly higher rate of positive anti-N IgG antibodies (91%). Anti-N IgG levels were significantly correlated with the number of prior COVID-19 infections (*p* = 0.015). Specifically, individuals with a history of four COVID-19 infections had higher anti-N IgG titers (14.1 ± 1.4) than those with only one experience of COVID-19 infection (9.4 ± 7.2). Individuals who were infected with COVID-19 after receiving the vaccine demonstrated higher levels of anti-N IgG, exhibiting a 25% increase in mean titer levels compared to those who were infected prior to vaccination. There was a statistically significant association between anti-N IgG positivity with age (*p* = 0.034), and smoking status (*p* = 0.006) of participants. Participants younger than 20 and older than 60 showed the highest positivity rate of anti-N (>90%). Smokers had a low positivity rate of anti-N (68.8%) compared to nonsmokers (83.6%). In conclusion, this study demonstrated that most COVID-19 vaccination recipients had positive IgG antibodies, with differences in the long-term immunoglobulin response depending on the type of vaccine administered and occurrence of COVID-19 infection.

## 1. Introduction

Since the emergence of SARS-CoV-2 in December 2019, there has been a significant global effort to investigate the virus known as COVID-19 and its clinical course [1]. The World Health Organization (WHO) declared COVID-19 a pandemic on 11 March 2020 [2], and since then, it has become evident that the development of a safe and effective vaccine is the most reliable way to mitigate its transmission [3]. In May 2023, the WHO withdrew the status of “pandemic” for COVID-19, although COVID-19 remains a global health threat [2].

Multiple COVID-19 vaccines have been developed, validated, approved, and administered globally. The mechanisms of action for COVID-19 vaccines include mRNA-based vaccines (Pfizer and Moderna), viral vector vaccines (AstraZeneca, Janssen Johnson & Johnson, and Sputnik V), protein subunit vaccine (Novavax), and inactivated virus vaccines (Sinopharm, Sinovac, and Coronovax) [2,3].

COVID-19 vaccines leverage different mechanisms to elicit both innate and adaptive immunity [4]. Adaptive immunity, which is characterized by its specificity and memory, plays an important role in neutralizing SARS-CoV-2 by generating antibodies through B cells [4]. These antibodies bind to the spike protein of the virus and prevent its entry into cells, thereby conferring immunity [1]. The resultant antibodies establish an immunological memory, which forms the basis of vaccine immunogenicity. Diagnostic tests for SARS-CoV-2 detect antibodies against nucleocapsid (N) and spike (S) antigens [4]. The S protein plays a crucial role in the viral attachment, entry, and fusion process [1]. It mediates the attachment of the virus to host cell surface receptors, allowing viral entry into the host cell [4]. Consequently, it is the primary protein targeted for developing COVID-19 therapeutic antibodies and vaccines [5]. On the other hand, the N protein primarily binds to the SARS-CoV-2 RNA genome, forming the nucleocapsid. Although N is mostly involved in processes related to the viral genome, it also plays a role in the viral assembly, budding, and host’s cellular response to viral infection [5].

Recent studies have aimed to elucidate the immunological mechanisms underlying the multifarious clinical outcomes observed in individuals infected with COVID-19 [6]. Notably, the presence of pathogen-specific antibodies is often indicative of protective immune mechanisms [7]. Numerous studies have been conducted to explore the factors responsible for recovery in some patients and severe complications in others [8]. It has been suggested that early humoral responses to SARS-CoV-2 antigens could play a consequential role in determining disease prognosis [9]. Specifically, it has been noted that patients who have recuperated from COVID-19 generated higher levels of anti-S-neutralizing antibodies, while those who experienced severe symptoms generated higher levels of anti-N antibodies [10].

The degree to which inactivated vaccines stimulate the production of anti-N antibodies in an immunized populace has not been thoroughly examined [11]. Few studies have investigated the presence of anti-N antibodies in the Sinopharm vaccine, but most have reported either the absence of such antibodies or detection in less than half of the cases analyzed [12]. Most COVID-19 vaccinations have been designed to induce the production of neutralizing anti-S antibodies, which prevent viral entry and facilitate virus clearance [11,13]. Compared to the Sinopharm vaccine, the Pfizer mRNA vaccine has demonstrated higher efficacy, as evidenced by a greater proportion of individuals developing neutralizing anti-S antibodies, suggesting a potential higher protection rate [13]. Regarding symptom severity and need for hospitalization, the Pfizer vaccine has shown a 95% efficacy rate in preventing future infections with SARS-CoV-2 [13]. In contrast, Sinopharm vaccine recipients exhibited a higher risk of post-vaccination infections, hospitalizations, ICU admission, and mortality than Pfizer recipients [14].

Consequently, the present study aims to investigate the long-term immunoglobulin response among individuals who have been vaccinated with either Sinopharm or Pfizer, using anti-S IgG, anti-N IgG, and IgM titers. Furthermore, this study intends to identify the underlying factors that account for the observed variations in titer levels across the respective groups including the role of natural COVID-19 infection.

## 2. Materials and Methods

### 2.1. Participants, Setting, and Ethical Consideration

A cross-sectional design was employed in this work. This study included the voluntary participation of 267 adult Jordanian individuals at Prince Hamza Hospital (PHH). This study was carried out from 28 July 2022 to 15 October 2022, during which participants completed a survey and provided serum samples. The serum samples were stored and analyzed for the presence of anti-S IgG, anti-N IgG, and anti-N IgM between 27 September and 2 November 2022. Serum antibody levels were evaluated 413.4 days (±135.3 days) from receiving the final dose. The study population was stratified into five groups, which were individuals with confirmed COVID-19 infection, and those who had either received a minimum one dose of Sinopharm, Pfizer, AstraZeneca, or Sputnik vaccines. The individuals with COVID-19 were confirmed to have the infection through a positive RT-PCR test conducted by an accredited laboratory. Data concerning sociodemographics and clinical history were collected for each participant. This study adhered to the guidelines of the Deceleration of Helsinki and was approved by the Institutional Review Board (IRB) committee at the Hashemite University (No.22/4/2021/2022) and PHH.

### 2.2. Demographic and Clinical Characteristics of Population Study

This study enlisted 267 individuals, consisting of those who were confirmed to have COVID-19 infection via PCR testing (*n* = 125), as well as Pfizer-vaccinated individuals (*n* = 133), Sinopharm-vaccinated individuals (*n* = 112), and individuals who received AstraZeneca (*n* = 20) or Sputnik (*n* = 2) vaccines from PHH.

### 2.3. Sample Collection

Serum specimens were obtained from study participants at PHH after obtaining their informed consent. Specimens of participants with confirmed COVID-19 infection were categorized based on the frequency of occurrence, as well as whether the infection occurred before or after the vaccination.

### 2.4. Sample Analysis

The automated Vitek Immuno Diagnostic Assay System (VIDAS®, Biomerieux Inc., Hazelwood, MO, USA) was used for the detection of IgG antibodies specific for SARS-CoV-2 in human serum using Enzyme Linked Fluorescent Assay (ELFA) technique. The assay combines a two-step sandwich enzyme immunoassay method with a final fluorescence detection. Anti-human IgG labeled with alkaline phosphatase was used for specific detection of IgG antibodies. The intensity of fluorescence is directly proportional to the level of antibody in the tested sample. An index is calculated as a ratio between the relative fluorescence value (RFV) measured in the sample and the RFV obtained for the calibrator (humanized recombinant anti-SARS CoV-2 IgG). The results were first interpreted as positive (index ≥ 1) or negative (index < 1), before being converted into binding antibody units per milliliter (BAU/mL) using a standard equation that comply with the WHO standard.

The COVID-19 IgG/IgM Duo assay was employed to quantitatively detect anti-N IgG and IgM in human serum through fluorescence immunoassay (FIA) using the FREND System of the same serum samples. The procedure was carried out in accordance with the protocol of NanoEntek. Samples exhibiting a concentration of less than 1.00 U/mL were deemed negative.

### 2.5. Statistical Analysis

Data were analyzed using Statistical Package for the Social Sciences (SPSS) version 25 (IBM, Chicago, IL, USA). Descriptive statistics were presented as frequencies and percentage or as mean and standard deviation (SD) as appropriate. Associations between anti-S or anti-N IgG positivity frequencies with COVID-19 vaccines, COVID-19 infection/s, and other demographic and clinical variables were tested using a chi-squared test or Fisher’s exact test. Differences in the mean titer of anti-S or anti-N IgG levels according to vaccine type or dose or according to number of COVID-19 infections or timing of infection were tested using Student’s *t*-test or one-way ANOVA followed by post-hoc testing with the LSD test when appropriate. *P* values were considered significant if ˂0.05. If there were less than 5 participants who received a certain vaccine (Sputnik, *n* = 2), they were excluded from inferential analysis.

## 3. Results

### 3.1. Participants (Demographics)

Overall, 267 participants with at least one dose of COVID-19 vaccination were included in this study. The demographic distribution of the selected individuals is shown in Table 1. Participants were 120 (44.9%) males and 147 (55.1%) females with most of the participants’ ages ranging between 20 and 60 years (76.4%). Most participants were nonsmokers (*n* = 173, 64.8%) and 156 (58.4%) had chronic diseases, with hypertension (27%) and diabetes (24.3%) being the most frequent chronic diseases. Less than a third (30.3%) of participants had a normal BMI, whereas 28.1% had a BMI over 30.

### 3.2. Participants’ Vaccination and COVID Infection History

**Figure 1:**
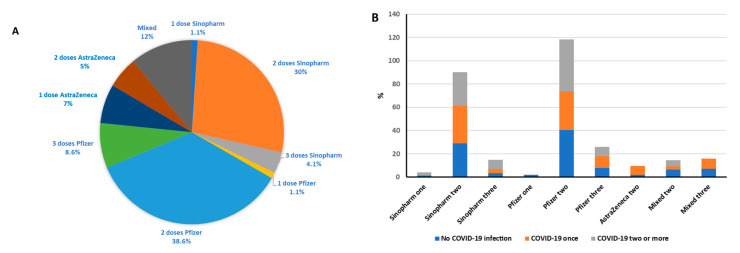
Percentages of COVID-19 vaccine types among patients (**A**) and percentages of COVID-19 infection among different COVID-19 vaccine types and doses (**B**).

Table 1 demonstrates that 260 (97.4%) participants received two or more doses of vaccination. Half of the participants received Pfizer as their first and second dose, while Sinopharm was taken by about 40% of the participants as their first two doses, and the rest received AstraZeneca and Sputnik vaccines. Moreover, 54 (20.2%) participants received an additional third dose with 8.6% of the sample receiving all three of these doses from Pfizer (Figure 1A). More than half (155, 58%) of the participants received their last dose of vaccination at least a year prior to participating in the survey while only six (2.2%) received their last dose less than 100 days before participating. COVID-19 infection was reported by 125 (46.8%) participants, with the majority (69.6%) of those who were infected having only one infection 84% were confirmed using RT-PCR, 30.4% were infected more than one time, and 69.6% were infected after vaccination. The distribution of COVID-19 infections according to vaccination type and dose are shown in Table 1 and Figure 1B. Participants who received COVID-19 vaccine/s and had COVID-19 infection/s before or after vaccination would have hybrid immunity (natural and vaccine induced).

### 3.3. Participants’ COVID-19 Immunoglobulin Responses to Vaccine

Most participants had positive IgG antibodies whether anti-S IgG (*n* = 257, 96.3%) or anti-N IgG (*n* = 202, 75.7%). Table 2 shows that participants’ anti-S IgG mean ± SD was (420.19 ± 213.65 BAU/mL) with 54.7% having titers above 400 BAU/mL, while only 16 (6.25%) participants had an anti-S IgG less than 100 BAU/mL out of those who had a positive response. On the other hand, Table 2 demonstrates that the mean ± SD level of anti-N IgG in participants was 9.36 ± 7.30 U/mL with only less than half of them (45.3%) having anti-N IgG above 10 U/mL. COVID-19 anti-N IgM antibodies according to Table 2 were negative in the majority (*n* = 241, 90.3%) of participants at the time of blood sample withdrawal, and the mean anti-N IgM of participants was 0.48 ± 0.65 U/mL.

### 3.4. COVID-19 Vaccine Effects on Antibodies

Analysis of the data presented in Table 3 shows statistically significant association between vaccination type/dose with anti-S (*p* = 0.02) and anti-N (*p* = 0.015) IgG antibody positivity frequency. For Sinopharm recipients, two doses had significantly lower frequency of positive anti-S IgG (93.8%) compared to two doses of Pfizer (93.8%), AstraZeneca (100%), or mixed (100%). Using the chi-squared test, all types of vaccines induced a 100% positive frequency of anti-S after the third dose (Table 3). Furthermore, participants with two and three doses of Sinopharm showed a significantly lower mean of anti-S IgG (323.0 ± 212.9 BAU/mL) compared to those with two or three doses of other vaccines (Pfizer, AstraZeneca, or mixed) as per one-way ANOVA analysis followed by post-hoc test using LSD.

On the contrary, two doses of Sinopharm had significantly higher frequency of positive anti-N IgG antibodies (91%) compared to other vaccines including Pfizer (72.7%), AstraZeneca (63.6%), or mixed vaccines (50.0%). Furthermore, Sinopharm had significantly higher mean of anti-N IgG titer compared to other vaccines. Unlike anti-S, the third dose of vaccination had less effect on increasing the anti-N positivity frequency among participants. Participants who received less than five doses of vaccination at the time of blood withdrawal were excluded from analysis.

### 3.5. COVID-19 Infection Effect on Anti-S IgG and Anti-N IgG Levels

Table 4 demonstrates the distribution of participants’ COVID-19 infection history and the timing of infection according to the date of their first dose of vaccination. Out of those with positive anti-N, 98 (48.5%) participants had no history of COVID-19 infection, while only 9 (4.5%) had three or more infections. Anti-N titers had a statistically significant association with the number of COVID-19 infections (*p* = 0.015). Participants with a history of four infections had a mean anti-N IgG titer of 14.1 ± 1.4, whereas those with one previous infection had a titer of 9.4 ± 7.2. The timing of infection also played a role in association with anti-N titers (*p* = 0.007). Participants who were infected after being vaccinated showed higher levels of anti-N at the time of blood sample withdrawal with a 25% higher anti-N mean titer compared to those infected before vaccination.

### 3.6. Effect of Hybrid Immunity on Anti-S IgG and Anti-N IgG Levels

The role of hybrid immunity (natural infection plus vaccination) in enhancing anti-S and anti-N antibodies levels compared to vaccination alone was investigated. As shown in Table 5, the mean anti-S IgG and anti-N IgG were significantly higher in hybrid immunity compared to vaccination alone among the whole study population (*p* < 0.05). Furthermore, among different vaccination types, anti-S IgG and anti-N IgG means were consistently higher among the hybrid immunity groups without achieving statistical significance.

### 3.7. Effect of Time on Anti-S IgG and Anti-N IgG Levels

**Figure 2:**
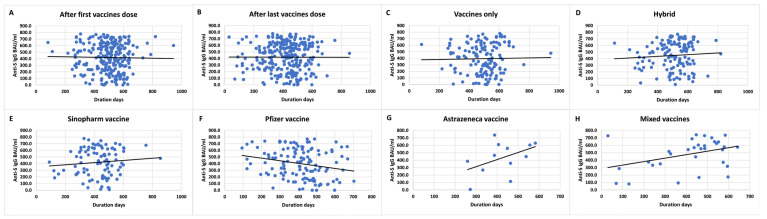
Longitudinal anti-S IgG titers according to vaccine doses: (**A**) first dose and (**B**) last dose; immunity type: (**C**) vaccine only and (**D**) hybrid; and vaccine type: (**E**) Sinopharm, (**F**) Pfizer, (**G**) AstraZeneca, and (**H**) mixed.

**Figure 3:**
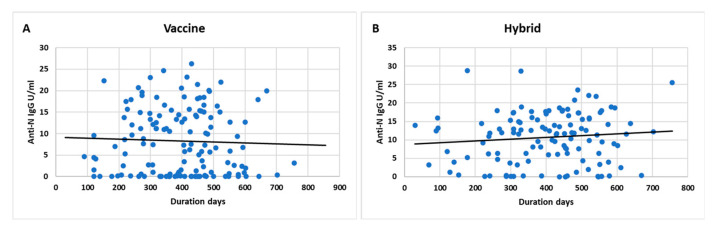
Longitudinal anti-N IgG titers over days according to immunity type (**A**) vaccine only and (**B**) hybrid.

The weaning effect of anti-S IgG titers over time indicated a stable antibody levels over time after the first dose or last dose of vaccination (Figure 2A,B). Anti-S antibodies increased slightly over time in hybrid immunity mostly due to the boosting effect of natural infection compared to vaccination alone (Figure 2C,D). A similar effect was noted with anti-N IgG antibodies showing increased levels over time with hybrid immunity compared to vaccination alone (Figure 3A,B). The weaning effect of anti-S IgG was more prominent with the Pfizer vaccine compared to other vaccine types (Figure 2E–H). A similar effect was observed with anti-N IgG levels for the Pfizer vaccine as well.

### 3.8. Effect of Age, Gender, BMI, Smoking, and Chronic Diseases on Anti-S IgG and Anti-N IgG Positivity and Titer Levels

Age, gender, BMI, and the smoking status of participants had no statistically significant effect on the positivity frequency of anti-S among participants (*p* > 0.05). However, as seen in Table 6, participants with comorbidities had a statistically significant (*p* = 0.044) lower positivity rate (148/155, 95.5%) of anti-S compared to those without chronic diseases (109/109, 100%). No significant differences in anti-S IgG titer mean within categories of age, gender, BMI, smoking or chronic diseases status were found (Table 6).

Meanwhile, although anti-N positivity showed statistically significant association with age (*p* = 0.034) and the smoking status (*p* = 0.006) of participants, there was no significant association with chronic diseases, BMI, or gender. Participants younger than 20 and those older than 60 had the highest positivity frequency of anti-N (>90%), while those between 20 to 40 and 41 to 60 had lower positivity rates of 70.1% and 77.7%, respectively. In addition to that, smokers had lower positivity frequency of anti-N (68.8%) than nonsmokers (83.6%). The mean anti-N IgG titer was also significantly higher in participants above 60 (12.0 ± 7.6 U/mL, *p* = 0.027) compared to other groups (Table 6).

Analysis of the effect of different variables including age, gender, BMI, smoking, and chronic disease status on anti-S IgG and anti-N IgG levels among different vaccine types (Sinopharm versus Pfizer) indicated no significant effect for all variables except age and anti-N IgG positivity and mean within the Pfizer vaccine group (Table 7).

## 4. Discussion

The COVID-19 vaccination rate has been increasing and most people have received at least two doses of COVID-19 vaccines with a coverage rate of 47.26% here in Jordan [15] and 81.3% in the United States [16]. Furthermore, with a good percentage of people receiving the third dose of the vaccine, it is important to precisely assess their immune response to the vaccines, especially the humoral antibody response. Antibody titer quantification for SARS-CoV-2 has been reported to be a good tool for classifying vaccinated people as known responders and low- or non-responders and for identifying if additional vaccination is needed to achieve good protection against COVID-19 [17].

Most tests that measure the serological state of COVID-19 depend on detecting antibodies to the N and S proteins, which are the most immunogenic parts of the virus. Many studies have investigated the quantitative value of anti-S and anti-N antibodies taking into consideration whether the subjects were infected or vaccinated [18,19,20]. The spike (S) protein present on the virus’s envelope plays a major role in the adsorption step and helps the virus enter human cells. Anti-S is a good indicator of an effective body response to the vaccine [21]. In contrast, nucleocapsid (N) protein is a part of the viral nucleoprotein complex and plays an important role in virus assembly, meaning that it plays an important role in natural infection [22,23]. The titer of anti-S and anti-N differs according to vaccination status, whether a previous infection occurred, and the severity of the symptoms. Therefore, it is important to detect the target antibody depending on the purpose of the test. When assessing the efficacy of the vaccine, anti-S antibodies play the more important role. In contrast, anti-N antibody has been regarded as more important when assessing the immune response to natural infection [20].

The third dose of the vaccine became recommended as a protective booster dose in most people aged 5 years or more, according to the Center for Disease Control; however, studies on vaccine efficacy after the third dose are lacking. There are a few studies that measure the quantity of anti-S and anti-N and compare their level in patients with and without previous infections [20]. As a result, in this study, we quantitatively compared the titer of the antibodies taking into consideration the status of the past infection, the type of vaccine, the number of doses, and the social characteristics of the subjects to know the factors playing an important role in vaccine response.

The three major types of antibodies studied were anti-S IgG, anti-N IgM, and anti-N IgG. Most of the study subjects were vaccinated (97.4%) with at least two doses, while only 46.8% of the subjects were infected, mostly one time. The most frequent seropositive antibody was for anti-S IgG (96.3%) with most of those having titer more than 400 BAU/mL. After that, anti-N IgG was 75.5%, with most of them below 10 U/mL, and the least was anti-N IgM (6%). These findings correlate with the characteristics of the subjects, as most of them were vaccinated, so the anti-S antibodies, which are the most important biomarker for vaccination, will be positive in most of the patients. However, only less than 50% were infected, so the anti-N IgG, which is the most important biomarker for a previous infection, was not positive at the same rate as anti-S antibodies [20].

The anti-S antibody titer was significantly higher after the second dose of Pfizer (99%) and AstraZeneca (100%) compared to Sinopharm (93.8%). This correlated with a finding from another study which found that the antibody response for Pfizer (99.4%) was higher than Sinopharm (71%) [24]. The subjects who received the third dose of any vaccine were 100% positive for anti-S antibodies, which confirms the importance of the third dose and its ability to boost the immune response and increase the longevity of the antibodies [25]. Nevertheless, the mean of the anti-S antibody titers was significantly lower in the Sinopharm group compared to Pfizer. The anti-N IgG antibody was positive in 75.5% of the subjects, which is much lower than the anti-S antibody positivity rate that coincides with other study findings [26]. An interesting finding was that among Sinopharm subjects, 91% were positive for anti-N IgG antibodies compared to Pfizer (72%) and AstraZeneca (63%) subjects. Also, the mean was higher in the Sinopharm group. This was mostly related to vaccination type and mechanism of action. The Pfizer vaccine (mRNA vaccine) and AstraZeneca vaccine (vector vaccine) both instruct cells to synthesize SAR-CoV-2 S protein and thus were expected to produce high levels of anti-S antibodies only. On the contrary, Sinopharm (inactivated vaccine), which contains both S and N proteins, was expected to induce high levels of anti-S and anti-N antibodies [11,12,13,20,21]. Positive anti-N antibodies in Pfizer and AstraZeneca vaccine recipients were mostly due to natural COVID-19 infections rather than vaccinations. Furthermore, the third dose vaccination had less capacity for increasing the anti-N positivity frequency compared to the anti-S antibody.

There was no significant association between the history of a previous infection and anti-S antibody titer consistent with what we discussed above—that the anti-S level was mostly boosted by the vaccine itself, not by the natural infection. There was a significant association between the timing of the infection and anti-S antibody titer being higher for subjects who became infected after the vaccine, which is mostly related to a shorter duration between the exposure and blood withdrawal for the study emphasizing the waning nature of the antibodies with time [27]. On the contrary, anti-N antibody positivity rate and titer mean were both significantly associated with the number and timing of COVID-19 infections with *p* values 0.032 and 0.015, respectively, and participants with a history of four infections had a mean anti-N IgG titer of 14.1 ± 1.4, whereas those with one previous infection had a titer of 9.4 ± 7.2. Furthermore, participants who were infected after being vaccinated showed higher levels of anti-N at the time of the blood sample withdrawal with a 25% higher anti-N mean titer compared to those infected before vaccination. This coincides with the same anti-S antibody finding strengthening the waning nature of both types of antibodies [28].

For anti-S antibodies, there was no statically significant association between positivity frequency and age, BMI, gender, and smoking status. However, there was a statically significant relationship between anti-S and participants with comorbidities, being a lower positivity rate (95.5%) compared to those with no chronic diseases (100%). This is consistent with the scientific findings that comorbidity weakens the immune system, which results in a lower capacity of antibody production [29]. On the contrary, anti-N IgG had a significant association with age and smoking status, but not with chronic disease status, meaning that participants with chronic diseases had the same positivity rate for anti-N IgG as chronic disease-free participants. Even though they have a relatively weak immune system, they suffer from severe COVID-19 infections, which makes the immune system fight a lot and increase the anti-N antibody level, which is mainly induced by natural infection, consistent with other study findings [29].

Similar studies on long-term anti-S and anti-N antibodies levels post-COVID-19 vaccination from North Africa and the Middle East region are summarized in Table 8. These studies indicated the superior ability of mRNA vaccines in inducing anti-S antibodies, higher ability of inactivated vaccines to produce anti-N antibodies, the role of a booster dose in enhancing antibody production, and significantly higher antibodies with past COVID-19 infection [30,31,32,33,34,35] in agreement with the findings of this study. Recent studies highlighted the increased long-term IgG4 levels of mRNA COVID-19 vaccines [36].

The main aims of this study were to analyze the humoral antibody response to available COVID-19 vaccines in Jordan, compare the common antibodies and their titer in response to different vaccines, and know the effect of a natural infection on antibody titers. Furthermore, it emphasized the effect of the third dose to confer a protective immune response against future infections. This finding might decrease public hesitancy toward the vaccines due to incorrect information appearing in recent times. The main limitation of this study was the different timing of blood withdrawal in relation to the last vaccine dose, which might return variable reads for the titers among participants. We recommend more studies to know the lifetime and subclass of the antibodies, which will provide us an indication for further doses of the vaccine.

## 5. Conclusions

In conclusion, this study demonstrated that most COVID-19 vaccination recipients had positive IgG antibodies, with differences in the long-term immunoglobulin response depending on the type of vaccine administered. Sinopharm-vaccinated individuals exhibited a lower rate of positive anti-S IgG, but a higher rate of positive anti-N IgG antibodies compared to other vaccines. This study also highlighted that prior COVID-19 infections may contribute to increased levels of anti-N IgG antibody generation. Additionally, patient age and smoking status have emerged as key factors in impacting the long-term immunoglobulin response. Overall, these findings have consequential implications for public health policies and vaccine development strategies. Further research in this area is required to enhance our understanding of the long-term immunoglobulin response to COVID-19 vaccination.

## Figures and Tables

**Table 1:** Demographic, clinical data and COVID-19 infection and vaccination details of study population (*n* = 267).

	Variable		Number (%)	
Age (years)	0–20 21–40 41–60 Above 60		11 (4.1) 97 (36.3) 107 (40.1) 52 (19.5)	
Gender	Male Female		120 (44.9) 147 (55.1)	
BMI	Underweight < 18.5 Normal 18.5–24.9 Overweight 25–29.9 Obese ≥ 30		9 (3.4) 81 (30.3) 102 (38.2) 75 (28.1)	
Smoking	Yes No		94 (35.2) 173 (64.8)	
Chronic diseases	Yes Hypertension Diabetes mellitus Cardiac diseases Hyperlipidemia Thyroid diseases Asthma Others (cancer, kidney diseases, digestive diseases, lung diseases)		156 (58.4) 72 (27.0) 65 (24.3) 39 (14.6) 34 (12.7) 35 (13.1) 13 (4.9) 46 (17.2)	
COVID-19 Infection	Yes Confirmed by RT-PCR One Two Three Four After vaccine Before vaccine		125 (46.8) 105 (39.3) 87 (32.6) 29 (10.9) 6 (2.2) 3 (1.1) 87 (32.6) 38 (14.2)	
COVID-19 Vaccination	Yes One dose Two doses Three doses Four doses		267 (100) 7 (2.6) 206 (77.2) 53 (19.9) 1 (0.4)	
Type of vaccine	Sinopharm Pfizer AstraZeneca Sputnik No	First dose 112 (41.9) 133 (49.8) 20 (7.5) 2 (0.7) 0 (0.0)	Second dose 108 (40.4) 135 (50.6) 16 (6.0) 1 (0.4) 7 (2.6)	Third dose 11 (4.1) 43 (16.1) 0 (0.0) 0 (0.0) 213 (79.8)
Duration (days) 0–100 101–200 201–300 301–400 401–500 Above 500 Not available	First dose 1 (0.4) 1 (0.4) 19 (7.1) 30 (11.2) 74 (27.7) 142 (53.2) -	Second dose 4 (1.5) 8 (3.0) 17 (6.4) 35 (13.1) 108 (40.4) 86 (32.2) 9 (3.4)	Third dose 2 (0.7) 2 (0.7) 17 (6.4) 18 (6.7) 2 (0.7) 2 (0.7) 224 (83.9)	Last dose 6 (2.2) 12 (4.5) 36 (13.5) 58 (21.7) 90 (33.7) 65 (24.3) -
		COVID-19 infection		
COVID-19 vaccine type and doses	Sinopharm one Sinopharm two Sinopharm three Pfizer one Pfizer two Pfizer three AstraZeneca two Mixed two Mixed three	No infection 2 (1.4) 41 (29.1) 5 (3.5) 3 (2.1) 57 (40.4) 11 (7.8) 3 (2.1) 9 (6.4) 10 (7.1)	Once 0 (0.0) 28 (32.2) 3 (3.4) 0 (0.0) 29 (33.3) 9 (10.3) 8 (7.6) 3 (2.9) 9 (8.6)	Two or more 1 (2.6) 11 (28.9) 3 (7.9) 0 (0.0) 17 (44.7) 3 (7.9) 0 (0.0) 1 (5.0) 0 (0.0)

**Table 2:** COVID-19 immunoglobulin response of study participants (*n* = 267).

Antibody	Variable	Category	Number (%) or Mean ± SD
Anti-S IgG antibodies	Number (%)	Positive Negative Not available	257 (96.3) 7 (2.6) 3 (1.1)
	Titer BAU/mL	Mean ± SD	420.19 ± 213.65
	Titer BAU/mL ranges	0–100 101–200 201–300 301–400 401–500 Above 500	23 (8.6) 32 (12.0) 28 (10.5) 35 (13.1) 35 (13.1) 111 (41.6)
Anti-N IgG antibodies	Number (%)	Positive Negative Not available	202 (75.7) 56 (21.0) 9 (3.4)
	Titer U/mL	Mean ± SD	9.36 ± 7.30
	Titer U/mL ranges	0–10 10.1–20 20.1–30	137 (51.3) 105 (39.3) 16 (6.0)
Anti-N IgM antibodies	Number (%)	Positive Negative Not available	16 (6.0) 241 (90.3) 10 (3.7)
	Titer U/mL	Mean ± SD	0.48 ± 0.65
	Titer U/mL ranges	˂1 ≥1	241 (90.3) 16 (6.0)

**Table 3:** Effect of COVID-19 vaccine type and doses on anti-S IgG and anti-N IgG levels.

		Sinopharm Doses		Pfizer Doses		AstraZeneca Doses	Mixed Doses		*p* Value
Antibody	Variable	2	3	2	3	2	2	3	
Anti-S IgG antibodies	Positive N (%)	75 (93.8)	11 (100)	99 (99)	23 (100)	11 (100)	13 (100)	18 (100)	0.020
	Negative N (%)	5 (71.4)	0 (0)	1 (14.3)	0 (0)	0 (0)	0 (0)	0 (0)	
	Titer BAU/mL Mean ± SD	323.0 ± 212.9	320.9 ± 182.4	470.4 ± 189.85	531.5 ± 220.1	437.8 ± 207.1	374.7 ± 179.3	489.3 ± 202.8	0.000
Anti-N IgG antibodies	Positive N (%)	71 (91)	9 (90)	72 (72.7)	19 (82.6)	7 (63.6)	6 (50)	13 (72.2)	0.015
	Negative N (%)	7 (12.7)	1 (1.8)	27 (49.1)	4 (7.3)	4 (7.3)	6 (10.9)	5 (9.1)	
	Titer U/mL Mean ± SD	10.9 ± 6.3	12.3 ± 7.9	8.3 ± 7.1	10.7 ± 8.0	9.4 ± 9.0	6.5 ± 8.1	7.5 ± 8.2	0.000

**Table 4:** Effect of COVID-19 infections number and timing on anti-S IgG and anti-N IgG levels.

		Number of COVID-19 Infections That Individuals Have Experienced						Timing of Infection		
Antibody	Variable	0	1	2	3	4	*p* Value	Before	After	*p* Value
Anti-S IgG antibodies	Positive N (%)	135 (52.5)	84 (32.7)	29 (11.3)	6 (2.3)	3 (1.2)	0.532	38 (14.8)	84 (32.7)	0.206
	Titer BAU/mL Mean ± SD	395.8 ± 218.9	420.7 ± 215.8	509.1 ± 168.1	469.9 ± 182.7	585.6 ± 67.4	0.060	393.2 ± 194.5	478.4 ± 203.2	0.009
Anti-N IgG antibodies	Positive N (%)	98 (48.5)	68 (33.7)	27 (13.4)	6 (3.0)	3 (1.5%)	0.032	28 (13.9)	76 (37.6)	0.012
	Titer U/mL Mean ± SD	8.3 ± 7.5	9.4 ± 7.2	12.5 ± 6.0	14.0 ± 3.4	14.1 ± 1.4	0.015	8.5 ± 5.9	11.3 ± 7.1	0.007

**Table 5:** Effect of hybrid immunity on anti-S IgG and anti-N IgG levels.

		Hybrid	Vaccine Only	*p* Value
All samples	Number	123	141	
	Anti-S IgG mean ± SD	448.04 ± 204.72	395.88 ± 218.99	0.048
	Anti-N IgG mean ± SD	10.55 ± 6.88	8.32 ± 7.52	0.014
Sinopharm	Number	45	49	
	Anti-S IgG mean ± SD	351.21 ± 206.94	307.68 ± 209.84	0.299
	Anti-N IgG mean ± SD	11.86 ± 5.44	10.44 ± 7.16	0.349
Pfizer	Number	57	70	
	Anti-S IgG mean ± SD	515.82 ± 175.19	449.92 ± 214.85	0.069
	Anti-N IgG mean ± SD	9.97 ± 7.23	7.66 ± 7.37	0.075
AstraZeneca	Number	8	4	
	Anti-S IgG mean ± SD	487.63 ± 202.26	392.82 ± 232.81	0.445
	Anti-N IgG mean ± SD	10.11 ± 8.34	5.68 ± 10.87	0.314
Mixed vaccines	Number	12	19	
	Anti-S IgG mean ± SD	488.63 ± 193.51	411.38 ± 201.16	0.302
	Anti-N IgG mean ± SD	9.44 ± 8.77	5.44 ± 7.30	0.131

**Table 6:** Effect of age, gender, BMI, smoking, and chronic diseases on anti-S IgG and anti-N IgG positivity and titer levels.

	Categories	Anti-S IgG			Anti-S IgG Titer	
		Positive N (%)	Negative N (%)	*p* Value	Mean ± SD	*p* Value
Age	0–20 21–40 41–60 Above 60	11 (4.3) 95 (37.0) 101 (39.3) 50 (19.5)	0 (0) 0 (0) 5 (71.4) 2 (28.6)	0.177	454.3 ± 219.4 414.3 ± 196.8 389.7 ± 210.2 485.7 ± 238.6	0.059
Gender	Male Female	116 (45.1) 141 (54.4)	3 (42.9) 4 (57.1)	1.000	436.2 ± 216.6 406.9 ± 210.9	0.269
BMI	Underweight < 18.5 Normal 18.5–24.9 Overweight 25–29.9 Obese ≥ 30	9 (3.5) 78 (30.4) 100 (38.9) 70 (27.2)	0 (0) 2 (28.6) 1 (14.3) 4 (57.1)	0.320	525.8 ± 184.6 425.6 ± 204.8 409.2 ± 215.5 416.2 ± 223.7	0.470
Smoking	Yes No	89 (34.6) 168 (65.4)	4 (57.1) 3 (42.9)	0.247	386.4 ± 197.5 438.5 ± 220.3	0.058
Chronic diseases	Yes No	148 (57.6) 109 (42.4)	7 (100.0) 0 (0)	0.044	420.7 ± 226.0 419.3 ± 195.7	0.957
		Anti-N IgG			Anti-N IgG titer	
	Categories	Positive N (%)	Negative N (%)	*p* Value	Mean ± SD	*p* Value
Age	0–20 21–40 41–60 Above 60	10 (5.0) 65 (32.2) 80 (39.6) 47 (23.3)	1 (1.8) 27 (48.2) 23 (41.1) 5 (8.9)	0.034	7.2 ± 6.6 8.5 ± 7.3 8.9 ± 6.9 12.0 ± 7.6	0.027
Gender	Male Female	94 (46.5) 108 (53.5)	25 (44.6) 31 (55.4)	0.802	9.5 ± 7.7 9.2 ± 6.9	0.734
BMI	Underweight < 18.5 Normal 18.5–24.9 Overweight 25–29.9 Obese ≥ 30	8 (4.0%) 57 (28.2) 78 (38.6) 59 (29.2)	1 (1.8) 20 (35.7) 20 (35.7) 15 (26.8)	0.658	10.3 ± 7.0 8.5 ± 7.1 9.6 ± 7.5 9.6 ± 7.2	0.726
Smoking	Yes No	64 (31.7) 138 (68.3)	29 (51.8) 27 (48.2)	0.006	8.3 ± 7.5 9.9 ± 7.1	0.100
Chronic diseases	Yes No	124 (61.4) 78 (38.6)	30 (53.6) 26 (46.4)	0.291	9.9 ± 7.5 8.4 ± 6.9	0.094

**Table 7:** Effect of age, gender, BMI, smoking, and chronic diseases on anti-S IgG and anti-N IgG positivity and titer levels among different vaccine types (Sinopharm and Pfizer).

Anti-S IgG				
	Sinopharm		Pfizer	
	Positivity (*p* value)	Titer (*p* value)	Positivity (*p* value)	Titer (*p* value)
Age	0.330	0.091	0.533	0.268
Gender	0.391	0.099	0.498	0.205
BMI	0.748	0.083	0.597	0.904
Smoking	0.169	0.075	1.00	0.468
Chronic diseases	0.079	0.253	0.505	0.445
Anti-N IgG				
	Sinopharm		Pfizer	
	Positivity (*p* value)	Titer (*p* value)	Positivity (*p* value)	Titer (*p* value)
Age	0.759	0.682	0.009	0.035
Gender	0.715	0.912	0.310	0.394
BMI	0.867	0.485	0.850	0.721
Smoking	0.104	0.968	0.096	0.383
Chronic diseases	1.00	0.465	0.148	0.072

**Table 8:** Studies reporting long-term anti-S and/or anti-N antibodies levels post-COVID-19 vaccination from North Africa and Middle East regions.

Country	Study Type	Study Population and Number	COVID-19 Vaccine Type	Study Goal	Major Conclusions	Citation
Qatar	Cross-sectional	Male participants of manual and craft worker population *n* = 300	Pfizer–BioNTech Moderna AstraZeneca Sinopharm Janssen Covaxin	Long-term anti-S and anti-N IgG antibodies titers	Participants vaccinated with mRNA vaccines had higher median anti-S IgG antibody titers. The median time to reach the lowest quartile was 3.53 months and 7.63 months for the non-mRNA vaccine recipients and Pfizer vaccine recipients, respectively.	[30]
UAE	Retrospective	Male expatriate workers *n* = 952	Sinopharm Sputnik V Pfizer–BioNTech	Anti-S, anti-N, and neutralizing IgG antibodies, and T-cell response	Priming or boosting with mRNA-based vaccines and with two or more doses was more potent for inducing high levels of humoral response	[31]
Iran	Cross-sectional	Healthcare workers *n* = 174	Oxford/AstraZeneca COVAXIN Sinopharm Sputnik V	To evaluate anti SARS-CoV-2 antibody response after the second dose of COVID-19 vaccine.	Anti-N and S antibodies mean levels were higher in adenoviral-vectored vaccines compared to inactivated virus vaccines. All antibody levels were significantly higher in those with a past COVID-19 infection	[32]
Jordan	Prospective observational	Random Jordanian adults *n* = 288	Sinopharm Pfizer–BioNTech	To compare anti-S antibodies in subjects vaccinated with Pfizer–BioNTech or Sinopharm vaccine	Fully vaccinated recipients of the Pfizer–BioNTech vaccine had superior quantitative efficiency compared to Sinopharm recipients	[33]
Jordan	Prospective observational	Random Jordanian adults *n* = 299	Sinopharm Pfizer–BioNTech	To compare the anti-N antibody levels in people vaccinated with Sinopharm or Pfizer’s or naturally infected unvaccinated adults	Inactivated virus vaccine, Sinopharm, induces an anti-N response that can boost that of natural infection or vice versa. On the other hand, the Pfizer mRNA-based vaccine induces a significantly stronger anti-S Ab response.	[34]
Morocco	Cross-sectional	Healthcare workers *n* = 82	AstraZeneca Sinopharm	To determine anti-S IgG levels five months after the second vaccination dose	No significant difference between the positivity rates of the vaccinated individuals for gender, age or vaccine type. Longevity of the anti-SARS-CoV-2 IgG antibodies at least five months after vaccination.	[35]

## Data Availability

Data are available as Supplementary Table S1.

## Supplementary Materials

The following supporting information can be downloaded at: https://www.mdpi.com/article/10.3390/vaccines11091398/s1, Table S1: Details of study participants.
